# Evaluation of plasma Alpha klotho levels in obese cases

**DOI:** 10.5937/jomb0-60664

**Published:** 2026-06-16

**Authors:** Ibrahim Solak, Ibrahim Guney, Nevin Sekmenli, Huseyin Kurku, Mehmet Ali Eryilmaz

**Affiliations:** 1 University of Health Sciences, Department of Family Medicine, Konya Beyhekim Health Application and Research Centre, Konya, Turkey; 2 Konya City Hospital, University of Health Sciences, Division of Nephrology, Department of Internal Medicine, Konya, Turkey; 3 University of Health Sciences, Department of Radiology, Konya City Hospital, Konya, Turkey; 4 University of Health Sciences, Department of Biochemistry, Konya City Hospital, Konya, Turkey; 5 University of Health Sciences, Department of General Surgery, Konya City Hospital, Konya, Turkey

**Keywords:** carotid intima media thickness, klotho, obesity, oxidative stress, debljina intime i medije karotidne arterije, kloto, gojaznost, oksidativni stres

## Abstract

**Background:**

Klotho is an anti-ageing gene that extends lifespan when overexpressed and accelerates ageing when disrupted. In our study, we will investigate the correlations between plasma <span style="color: rgb(32, 33, 34); font-family: sans-serif; font-size: 16px; background-color: rgb(248, 249, 250);">α</span>-Klotho (<span style="color: rgb(32, 33, 34); font-family: sans-serif; font-size: 16px; background-color: rgb(248, 249, 250);">α</span>-KL) protein levels with body mass index (BMI), carotid intima media thickness (CIMT), total antioxidant status (TAS), total oxidant status (TOS), oxidative stress index (OSI), high-sensitivity C-reactive protein (hsCRP) and cholesterol values in obese patients.

**Methods:**

This study was conducted as a single-centre, cross-sectional study.

**Results:**

The study included 90 participants: 35 women (38.9%) and 55 men (61.1%). Of the participants included in the study, 30 were normal weight (33.3%) (Group 1), 30 were overweight (33.3%) (Group 2), and 30 were obese (33.3%) (Group 3). There were statistically significant differences in the <span style="color: rgb(32, 33, 34); font-family: sans-serif; font-size: 16px; background-color: rgb(248, 249, 250);">α</span>-KL protein (p = 0.010), hsCRP (p&lt; 0.001), and homeostasis model assessment of insulin resistance (HOMA-IR) (p = 0.022) between the study groups. There is no significant difference between the study groups for CIMT (p= 0.380), TOS (p= 0.613), TAS (p= 0.056), or OSI (p= 0.943).

**Conclusions:**

In this study, the overweight group had a-KL protein levels that were significantly higher than those in the obese group, whereas hsCRP levels were significantly higher in the obese group than in the overweight and normal weight groups. There were no significant differences in CIMT, TAS, TOS, and OSI values between the study groups. There were no significant correlations between <span style="color: rgb(32, 33, 34); font-family: sans-serif; font-size: 16px; background-color: rgb(248, 249, 250);">α</span>-KL protein values and BMI, hsCRP CIMT, TAS, TOS, or OSI in participants in the study.

## Abstract (sr)

**Uvod:**

Kloto "anti-age" GEN je GEN koji produžava životni vek kada je prekomerno eksprimiran, a ubrzava starenje kada njegova funkcija poremećena. U ovoj studiji istraživaćemo povezanost između nivoa <span style="color: rgb(32, 33, 34); font-family: sans-serif; font-size: 16px; background-color: rgb(248, 249, 250);">α</span>-Klotho (<span style="color: rgb(32, 33, 34); font-family: sans-serif; font-size: 16px; background-color: rgb(248, 249, 250);">α</span>-KL) proteina u plazmi i indeksa telesne mase (BMI), debljine intime i medije karotidne arterije (CIMT), ukupnog antioksidativnog statusa (TAS), ukupnog oksidativnog statusa (TOS), indeksa oksidativnog stresa (OSI), visokoosetljivog C-reaktivnog proteina (hsCRP) i vrednosti holesterola kod gojaznih pacijenata.

**Metode:**

Ova studija je sprovedena kao jednocentrična studija preseka.

**Rezultati:**

U studiju je bilo uključeno 90 ispitanika: 35 žena (38,9%) i 55 muskaraca (61,1%). Od ukupnog broja ispitanika, 30 je imalo normalnu telesnu težinu (33,3%) (Grupa 1), 30 je bilo prekomerne težine (33,3%) (Grupa 2), a 30 gojazno (33,3%) (Grupa 3). Statistički značajne razlike između grupa utvrđene su u vrednostima <span style="color: rgb(32, 33, 34); font-family: sans-serif; font-size: 16px; background-color: rgb(248, 249, 250);">α</span>-KL proteina (p= 0,010), hsCRP-a (p&lt; 0,001) i indeksa insulinske rezistencije prema modelu homeostaze (HOMA-IR) (p= 0,022). Nije bilo značajnih razlika između grupa u pogledu CIMT-a (p= 0,380), TOS-a (p= 0,613), TAS-a (p= 0,056) i OSI-ja (p= 0,943).

**Zaključak:**

U ovoj studiji, grupa sa prekomernom težinom je imala značajno više vrednosti <span style="color: rgb(32, 33, 34); font-family: sans-serif; font-size: 16px; background-color: rgb(248, 249, 250);">α</span>-KL proteina u poređenju sa grupom gojaznih, dok su vrednosti hsCRP-a bile značajno više u grupi gojaznih nego u grupama sa normalnom težinom. Nije bilo značajnih razlika između grupa u vrednostima CIMT-a, TAS-a, TOS-a i OSI-ja. Takođe, nisu uočene značajne korelacije između vrednosti <span style="color: rgb(32, 33, 34); font-family: sans-serif; font-size: 16px; background-color: rgb(248, 249, 250);">α</span>-KL proteina i BMI-a, hsCRP-a, CIMT-a, TAS-a, TOS-a ili OSI-ja kod ispitanika u studiji.

## Introduction

Being overweight and obese is defined as an abnormal or excessive accumulation of fat that poses health risks. According to body mass index (BMI), values above 25 indicate overweight, and values of 30 or higher indicate obesity. The prevalence of overweight and obesity continues to rise in both adults and children. From 1990 to 2022, the percentage of children and adolescents aged 5-19 years with obesity worldwide increased fourfold, from 2% to 8%, while the percentage of adults aged 18 years and older with obesity more than doubled, from 7% to 16%. In 2019, an estimated 5 million deaths from non-infectious diseases were attributed to a higher BMI than optimal [1]. Obesity is rapidly increasing globally and is regarded as a pandemic.

Obesity increases the risk of hypertension, type II diabetes mellitus and coronary heart disease. Obesity is associated with colorectal, pancreatic and renal cancers [2]. Obesity is a serious public health problem due to its association with diseases.

Metabolic changes associated with obesity are similar to changes observed during normal ageing. New evidence suggests that obesity may accelerate ageing [3].

Diseases related to obesity appear to be linked to the acceleration of cellular processes observed during normal ageing [4]. Inflammation and oxidative stress seem to be important mediators of this relationship.

Variable metabolic regulation, insulin resistance, inflammation, and disrupted immune function are distinguishing features not only of obesity but also of ageing. Interestingly, fat tissue in obese and elderly individuals displays notably similar immunological profiles, suggesting that the mechanisms directing both processes involve significant overlap [5].

Klotho is defined as a new gene involved in the suppression of several ageing phenotypes. The product of the Klotho gene may act as part of a signalling pathway regulating in vivo ageing and morbidity in age-related diseases [6]. Klotho is an anti-ageing gene that extends lifespan when overexpressed and accelerates ageing when disrupted [6].

α-Klotho (α-KL) may suppress insulin signalling and contribute to the pathogenesis of insulin resistance [7][8]. Animal experiments by Saito et al. [8] reported that adenovirus-mediated delivery of the Klotho gene may improve vascular endothelial dysfunction, increase nitric oxide production, lower blood pressure, and prevent medial hypertrophy and perivascular fibrosis in subjects with multiple atherosclerotic risk factors (hypertension, hypertriglyceridemia, hypoglycemia, and obesity) [8].

α-KL protein is mainly expressed in the kidneys, parathyroid glands and the choroid plexus in the brain. Still, it is also represented at lower levels by other organs, including the liver, skeletal muscles, fat tissue and the placenta [6][9].

Although most studies have focused on the role of the α-KL protein in calcium and phosphorus homeostasis, there is evidence that it confers resistance to oxidative stress at the cellular and organism levels [10][11]. α-KL protein may confer resistance to oxidative stress by activating the AMP signalling pathway, increasing superoxide dismutase expression, and inducing nitric oxide production [11].

α-KL protein was also shown to have anti-inflammatory activity [12][13].

Atherosclerosis is the underlying cause of most cardiovascular events. Atherosclerosis is a commonly observed inflammatory disease characterised by the accumulation of lipids and inflammatory cells and the development of scar tissue covered by a fibrous cap within the walls of large- and medium-sized arteries [14].

One of the earliest stages of atherosclerosis is arterial wall thickening. Carotid intima-media thickness (CIMT) measured by B-mode ultrasound is frequently used for noninvasive assessment of individuals at risk of atherosclerosis.

In our study, we investigated the correlations between plasma α-KL protein levels and BMI, CIMT, total antioxidant status (TAS), total oxidant status (TOS), oxidative stress index (OSI), high-sensitivity C-reactive protein (hsCRP), and cholesterol in obese patients.

## Materials and methods

This study received approval from the University Non-Drug and Medical Device Research Ethics Committee dated 18 June 2019, No. 2019/0044. It was conducted from August 1, 209 to August 31, 2019 at the Internal Medicine and Obesity Clinics of the Education and Research Hospital. Participants attending these clinics were informed about the study. Those who agreed to participate, did not meet any exclusion criteria, and signed an informed consent form in accordance with the World Medical Association Helsinki Declaration were included.

### Exclusion criteria

1 - Those who had any infectious disease in the last month, those diagnosed with diabetes mellitus, cardiovascular disease, cancer, gastrointestinal, pulmonary, renal or neurological disease,

2 - Those receiving steroid and immunosuppressive treatment,

3 - Smokers were not included in the study.

Blood from participants was collected in routine biochemistry tubes (gel-vacuum) as 5 mL venous blood samples from 08.00-12.00 in the morning, after 10-12 hours of fasting. Samples were centrifuged at 3000 g for 10 minutes. Serum of the samples was separated. Separated serum samples were stored at -80°C until the study. The samples were stored at this temperature for 45 days. On the day of the study, samples were brought to room temperature and studied on the same day. From samples, glucose, triglyceride (TG), total cholesterol (TC), high-density lipoprotein cholesterol (HDL-C), low-density lipoprotein cholesterol (LDL-C), insulin, hsCRP, α-KL, TAS and TOS were studied.

Glucose, TC, HDL-C, TG, and LDL-C were measured enzymatically using manufacturer-supplied kits on a Beckman Coulter AU 5800 (Beckman Coulter, Inc., CA 92821, USA). Insulin was measured by direct chemiluminescence on a Siemens Immulite 2000 (USA) device. hsCRP was measured nephelometrically with a Siemens hsCRP commercial kit (Siemens Healthcare Diagnostics, Marburg, Germany) on an NFL BN-II device (Dade Behring Diagnostics, Marburg, Germany).

TAS and TOS tests and OSI calculation: TOS and TAS commercial kits (Rel Assay Diagnostics, Megatip Sanayi ve Ticaret Ltd. ti., Gaziantep, TÜRKiYE) were applied to an Abbott Architect C16000 (Abbott Laboratories, Abbott Park, IL, USA) clinical chemistry autoanalyser, and samples were analysed. The TOS values of samples were proportioned to TAS values in percentages, and the OSI value was calculated:

OSI (arbitrary unit, AU) = ((TOS, μmolH2O2 eq/L)/(TAS, μmolTroloxeq/L)*100) [15].

The α-KL protein levels in serum samples were measured with a human α-KL ELISA kit (Cat. No: E4142Hu, Sensitivity: 0.021 ng/mL, Intra-Assay CV: <8%, Inter-Assay CV: <10%, Bioassay Technology Laboratory, 1008 Junjiang Inter. Bldg 228 Ningguo Rd, Yangpu Dist, Shanghai, China).

All ultrasound investigations were performed with the same ultrasound device (Aplio 500, Toshiba Medical System Corporation, Tokyo, Japan) using high-frequency (4-14 MHz) linear converter and by a single radiologist.The homeostasis model assessment of insulin resistance (HOMA-IR) values of participants were calculated with the following formula:

HOMA-IR=[Fasting insulin (microU/mL)xFasting glucose (mg/dl)/405]

Participants in the study were divided into 3 groups according to BMI. Those with BMI <25.0 were normal weight (Group 1), those with BMI 25.0-29.9 were overweight (Group 2), and those with BMI 30.0 were considered obese (Group 3).

### Statistical analysis

Descriptive analyses of categorical data in the study are presented as frequencies and percentages, while numerical data are reported as mean ± standard error. The assumption of normality for the data in the study groups was evaluated using the Shapiro-Wilk and Kolmogorov-Smirnov tests. Comparison of data without normal distribution between the study groups was performed using the Wilcoxon test and the Kruskal-Wallis test. Significance values for multiple tests were set with the Bonferroni correction. Comparisons of categorical data in the study used the chi-square test. The correlations between alpha-klotho and other participant parameters were evaluated using Spearman's correlation analysis. A binary logistic regression model was developed to identify independent predictors of obesity (BMI 30). To assess the predictive properties of α-KL and TG values by BMI category (BMI 30: obesity) and to determine cut-off values, ROC analysis was performed. All statistical analyses used in the study were two-tailed, with a 5% significance level and a 95% confidence interval. All statistical analyses were completed using SPSS 22.0 (IBM Inc., Armonk, NY, USA) software.

## Results

The study included 90 participants: 35 women (38.9%) and 55 men (61.1%). Of the participants included in the study, 30 were normal weight (33.3%) (Group 1), 30 were overweight (33.3%) (Group 2), and 30 were obese (33.3%) (Group 3).

The mean age of participants in the study was 36.45±1.01 years (min = 26, max=48). The comparison of sociodemographic and biochemical parameters in the groups is shown in Table 1.

**Table 1:** Comparison of parameters in the study groups. CIMT, carotid intima-media thickness; a-KL, a-Klotho; TOS, total oxidant status; TAS, total antioxidant status; OSI, oxidative stress index; hsCRP high-sensitivity C-reactive protein; TC, total cholesterol; TG, triglyceride; HDL-C, high-density lipoprotein cholesterol; LDL-C, low-density lipoprotein cholesterol; HOMA-IR, homeostasis model assessment of insulin resistance.

		Group 1 <br>n=30 (33.3%)	Group 2 <br>n=30 (33.3%)	Group 3 <br>n=30 (33.3%)	p
Sex	Woman	13 (43.3%)	12 (40.0%)	10 (33.3%)	0.721
	Man	17 (56.7%)	18(60.0%)	20(66.7%)	
Age (years)		35.44±1.71	36.33±1.30	37.94±1.96	0.124
CIMT (mm)		0.63±0.02	0.65±0.02	0.68±0.04	0.380
a-KL protein (pg/mL)		3.75±0.38	4.25±0.39	2.68±0.34	0.010
TOS (μmol H_2_O_2_ Eq/L)		49.13±6.59	65.57±10.42	64.59±10.80	0.613
TAS (μmol-Trolox Eq/mL)		1.33±0.05	1.35±0.06	1.43±0.06	0.056
OSI		3.71±0.49	4.75±0.67	4.55±0.74	0.943
hsCRP (mg/L)		2.63±0.44	4.70±1.20	5.01±0.73	<0.001
Glucose (mg/dL)		89.89±3.96	96.57±4.08	93.00±3.53	0.382
TC (mg/dL)		198.06±8.35	200.67±5.97	207.12±9.45	0.404
TG (mg/dL)		147.72±16.78	179.57±27.02	227.71±32.86	0.294
LDL-C (mg/dL)		108.33±9.85	120.90±5.98	122.71±9.37	0.568
HDL-C (mg/dL)		40.11±3.28	43.90±2.34	40.88±1.95	0.974
Insulin (μIU/mL)		13.04±4.09	22.76±5.49	24.71±6.81	0.010
HOMA-IR		3.44±1.47	6.11±1.72	5.95±1.68	0.022

A statistically significant difference in α-KL protein levels was observed among the study groups (p=0.010). When the significance level for multiple testing is adjusted using Bonferroni corrections, there are no significant differences between Group 1 and Group 2, and between Group 1 and Group 3 (p=0.440, p=0.313, respectively). However, a significant difference remains between Group 2 and Group 3 (p=0.007).

A statistically significant difference in hsCRP levels was observed among the study groups (p<0.001). When the significance value for multiple tests is set with the Bonferroni correction, there were significant differences between Group 1 and Group 3 and between Group 2 and Group 3 (p<0.001, p = 0.035, respectively), with no significant difference between Group 1 and Group 2 (p=0.147).

A statistically significant difference in insulin levels was observed among the study groups (p=0.010). When the significance value for multiple tests is set with the Bonferroni correction, there were no significant differences between Group 1 and Group 2 and between Group 2 and Group 3 (p=0.070, p=1.000, respectively). At the same time, there was a significant difference between Group 1 and Group 3 (p=0.012).

A statistically significant difference in HOMA-IR levels was observed among the study groups (p=0.022). When the significance value for multiple tests is adjusted with the Bonferroni correction, there were no significant differences between Group 1 and Group 2, or between Group 2 and Group 3 (p=0.139, p=1.000, respectively). Still, there was a significant difference between Group 1 and Group 3 (p=0.023).

There was a significant positive correlation between α-KL protein and TC in participants in the study (r=0.259, p = 0.021). The correlations between α-KL protein and other parameters for participants in the study are shown in Table 2.

**Table 2:** Correlation of α-KL protein with other parameters for participants in the study. CIMT, carotid intima-media thickness; a-KL, a-Klotho; TOS, total oxidant status; TAS, total antioxidant status; OSI, oxidative stress index; hsCRP high-sensitivity C-reactive protein; TC, total cholesterol; TG, triglyceride; HDL-C, high-density lipoprotein cholesterol; LDL-C, low-density lipoprotein cholesterol; HOMA-IR, homeostasis model assessment of insulin resistance.

	r	p
Age (years)	0.003	0.976
CIMT (mm)	-0.110	0.399
BMI (kg/m^2^)	-0.164	0.154
TOS (μmol H_2_O_2_ Eq/L)	0.058	0.613
TAS (μmol-Trolox Eq/mL)	0.098	0.383
OSI	0.049	0.671
hsCRP (mg/L)	-0.200	0.074
Glucose (mg/dL)	0.026	0.823
TC (mg/dL)	0.259	0.021
TG (mg/dL)	0.200	0.075
LDL-C (mg/dL)	-0.003	0.981
HDL-C (mg/dL)	0.100	0.380
Insulin (μIU/mL)	-0.032	0.777
HOMA-IR	-0.008	0.942

The TOS (p=0.002), OSI (p=0.001), and HDL-C (p<0.001) values of women participants in the study were statistically significantly higher. In contrast, the TAS (p<0.001) and TG (p<0.001) values of men participants were statistically significantly higher. The comparison of parameters by participant sex is shown in Table 3.

**Table 3:** Comparison of parameters according to sex. CIMT, carotid intima-media thickness; a-KL, a-Klotho; TOS, total oxidant status; TAS, total antioxidant status; OSI, oxidative stress index; hsCRP high-sensitivity C-reactive protein; TC, total cholesterol; TG, triglyceride; HDL-C, high-density lipoprotein cholesterol; LDL-C, low-density lipoprotein cholesterol; HOMA-IR, homeostasis model assessment of insulin resistance.

	Womenn=35 (38.9%)	Menn = 55 (61.1%)	p
a-KL protein (pg/mL)	3.28±0.31	3.84±0.32	0.434
Age (years)	36.13±1.20	36.81±1.36	0.540
BMI (kg/m^2^)	26.53±1.12	28.11±1.17	0.564
CIMT (mm)	0.63±0.02	0.67±0.02	0.327
TOS (μmol H_2_O_2_ Eq/L)	69.73±9.25	53.20±6.65	0.002
TAS (μmol-Trolox Eq/mL)	1.25±0.06	1.45±0.03	<0.001
OSI	5.37±0.60	3.64±0.44	0.001
hsCRP (mg/L)	4.96±1.11	3.55±0.46	0.230
Glucose (mg/dL)	91.91±2.86	94.33±3.29	0.688
TC (mg/dL)	202.65±6.80	201.18±5.99	0.793
TG (mg/dL)	122.30±9.56	226.91±22.88	<0.001
LDL-C (mg/dL)	128.65±5.90	109.58±6.76	0.290
HDL-C (mg/dL)	46.70±2.36	38.33±1.69	<0.001
Insulin (μIU/mL)	15.32±3.71	23.65±4.76	0.044
HOMA-IR	3.75±10.4	6.22±1.41	0.092

In the binary logistic regression model, α-KL (Beta: -1.068; SE: 0.383; 95% CI: 0.162-0.728; p=0.005) and TG (Beta: 0.010; SE: 0.004; 95% CI: 1.001-1.019; p=0.027) were identified as independent determinants of obesity (BMI 30) (Table 4).

**Table 4:** Independent determinants of obesity (BMI≥30). p=0.003; Nagelkerker's R[=0.504; Hosmer and Lemeshow test= 0.829

Variable	*β*	*SE*	95% Cl		*P*
			*LL*	*UL*	
CIMT	1.297	2.766	0.016	828.044	0.639
α-KL	-1.068	0.383	0.162	0.728	0.005
Age	0.118	0.070	0.982	1.290	0.089
OS	0.018	0.165	0.736	1.408	0.914
hsCRP	0.170	0.111	0.953	1.474	0.127
TG	0.010	0.004	1.001	1.019	0.027
LDL-C	0.026	0.015	0.997	1.056	0.076
HOMA-IR	0.000	0.069	0.874	1.145	0.999
Gender	-0.926	1.015	0.054	2.895	0.361
Constant	-8.658	3.613			0.017

**Figure 1:**
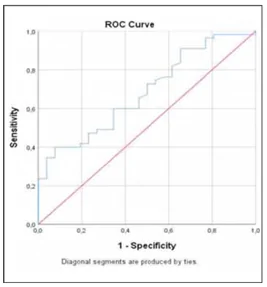
ROC curve demonstrating the predictive value of α-KL levels for obesity (BM≥30).

ROC analyses demonstrated that α-KL levels had predictive value for obesity (BMI 30), whereas TG levels did not (Figure 1). Accordingly, the calculated AUC for α-KL was 0.585 (SE: 0.065; 95% CI: 0.565-0.804; p=0.008), and the cut-off value of α-KL=3.138 predicted obesity with 60.0% sensitivity and 65.4% specificity.

## Discussion

In this study, an attempt was made to show the correlations between the anti-ageing gene product, α-Klotho protein, and obesity, inflammation, oxidative stress, and CIMT [15].

There is variable information in the literature about differences in the serum α-KL protein concentrations between the sexes. Pedersen et al. [16] reported that women had higher α-KL protein levels, whereas Yamazaki et al. [17] found no difference in α-KL protein levels between women and men. In our study, partly consistent with the literature, no significant difference was identified between women and men participants.

Several studies report a negative correlation between α-KL protein and age [16][18]. In this study, no correlation was found between participants' ages and klotho levels. The clustering of participants' ages within a certain interval in our study may have caused this.

A systematic review by Stikn CH van den Oord et al. [19] reported a positive correlation between the increase in CIMT and cardiovascular risk. Imad Abdullah et al. [20] noted an independent correlation between CIMT and α-KL protein, with low α-KL protein levels associated with increased CIMT In all studies examining the correlation between α-KL protein and CIMT, participants had a condition that increased CIMT No studies on CIMT and α-KL protein in healthy participants were identified in the literature. In our study, we did not find a significant relationship between CIMT and klotho. This may be because participants in our study did not have any comorbidities that could influence CIMT values.

Several clinical studies have shown that the α-KL protein has strong cardioprotective effects. For example, while α-KL protein was shown to protect against vascular calcification in rodent models of chronic renal failure (CRF), higher α-KL protein levels in people without CRF were associated with a lower mortality rate and cardiovascular disease (CVD) [20][21].

Though the mean CIMT in the overweight group was higher than in the normal group, and the obese group had a higher mean CIMT than the overweight group, the difference between the groups was not statistically significant. One reason may be the small number of participants in the study. Another reason may be that participants in the study were young people.

In the literature, contradictory results have been reported regarding obesity and α-KL. A study by Orces et al. [22] found that general and abdominal obesity in women were inversely associated with serum α-KL protein levels, and that obese women with at least the last decade of obesity had continuously lower serum α-KL protein levels than non-obese peers. The study reported that weight differences did not affect α-KL protein levels in men [22]. A study by Zelazniewicz et al. [23] in healthy men reported no correlation between BMI and α-KL protein values. A study by Amitani et al. [24] found low α-KL protein values in the groups of thin patients with anorexia nervosa and healthy obese participants compared to the control group. In this study, although the α-KL protein values of obese participants were significantly lower than those of overweight participants, no significant difference was found between the α-KL protein values of normal weight and overweight participants, or between those of normal weight and obese participants.

Once again, the literature reports variable results regarding the correlations between α-KL protein and hsCRP levels. Solak et al. [25] did not observe a correlation between α-KL protein and hsCRP in the non-smoking group, despite higher levels of both in the smoking group compared to the non-smoking group. A study by Zelazniewicz et al. [23] involving healthy men found no correlation between α-KL protein and hsCRP. Meanwhile, a study by Martfn-Nunez et al. [26] on patients with atherosclerosis examined inflammatory markers such as TNF-α, IL-6, and IL-10. This research reported an inverse correlation between α-KL protein and inflammation in patients with atherosclerosis. Jia et al. [27] identified a negative correlation between the systemic inflammatory index and α-KL protein in patients with albuminuria, and noted that higher α-KL protein levels were protective against systemic inflammation. Consistent with the existing literature, this study found no significant correlation between α-KL protein and hsCRP levels among participants.

In a study, no correlation was found between α-KL protein and TAS, TOS, and OSI in a healthy, non-smoking group [25]. In the study groups, there was no significant difference between TAS, TOS and OSI values. No significant correlation was found among participants in the study between α-KL protein, TAS, TOS, and OSI values.

## Conclusions

In conclusion, this study found that the overweight group had significantly higher α-KL protein levels compared to the obese group. Conversely, hsCRP levels were significantly higher in the obese group than in the overweight and normal weight groups. No significant differences were observed in CIMT, TAS, TOS, and OSI values among the study groups. Although there were no significant correlations between α-KL protein levels and BMI, hsCRP CIMT, TAS, TOS, and OSI, a weak positive correlation with TC was noted. Binary logistic regression identified α-KL and TG levels as independent predictors of obesity (BMI 30). ROC analysis demonstrated that α-KL measurements possessed significant predictive value for obesity, whereas TG measurements did not.

## Dodatak

### Limitations

There are some significant limitations to this study. As the study was cross-sectional, causality between α-KL protein and obesity could not be determined. Participants were not asked how long they had been at their weight during the study. Given the low number of participants, our data cannot be generalised to the whole society. There is a need for larger prospective studies with more participants about this topic.

### Funding

None.

### Authors' contribution

Conceptualisation and data collection: IS, NS HK. Research design and statistical analysis: IS, IG. Drafting of the original manuscript: IS, IG. Revision of the final manuscript: IS, IG, nS, h K and MAE. All authors agreed to be accountable for all aspects of the work, ensuring that questions related to the accuracy or integrity of any part of the study are appropriately investigated and resolved. All authors edited and approved the final version of the manuscript.

### Conflict of interest statement

All the authors declare that they have no conflict of interest in this work.
